# Activated Platelets in Carotid Artery Thrombosis in Mice Can Be Selectively Targeted with a Radiolabeled Single-Chain Antibody

**DOI:** 10.1371/journal.pone.0018446

**Published:** 2011-03-30

**Authors:** Timo Heidt, Friederike Deininger, Karlheinz Peter, Jürgen Goldschmidt, Annette Pethe, Christoph E. Hagemeyer, Irene Neudorfer, Andreas Zirlik, Wolfgang A. Weber, Christoph Bode, Philipp T. Meyer, Martin Behe, Constantin von zur Mühlen

**Affiliations:** 1 Department of Cardiology and Angiology, University of Freiburg, Freiburg, Germany; 2 Department of Nuclear Medicine, University of Freiburg, Freiburg, Germany; 3 Baker IDI Heart and Diabetes Institute, Melbourne, Australia; 4 Leibniz-Institute for Neurobiology, Magdeburg, Germany; 5 Department of Nuclear Medicine, University of Magdeburg, Magdeburg, Germany; Genentech, United States of America

## Abstract

**Background:**

Activated platelets can be found on the surface of inflamed, rupture-prone and ruptured plaques as well as in intravascular thrombosis. They are key players in thrombosis and atherosclerosis. In this study we describe the construction of a radiolabeled single-chain antibody targeting the LIBS-epitope of activated platelets to selectively depict platelet activation and wall-adherent non-occlusive thrombosis in a mouse model with nuclear imaging using *in vitro* and *ex vivo* autoradiography as well as small animal SPECT-CT for *in vivo* analysis.

**Methodology/Principal Findings:**

LIBS as well as an unspecific control single-chain antibody were labeled with ^111^Indium (^111^In) via bifunctional DTPA ( = ^111^In-LIBS/^111^In-control). Autoradiography after incubation with ^111^In-LIBS on activated platelets *in vitro* (mean 3866±28 DLU/mm^2^, 4010±630 DLU/mm^2^ and 4520±293 DLU/mm^2^) produced a significantly higher ligand uptake compared to ^111^In-control (2101±76 DLU/mm^2^, 1181±96 DLU/mm^2^ and 1866±246 DLU/mm^2^) indicating a specific binding to activated platelets; *P*<0.05. Applying these findings to an *ex vivo* mouse model of carotid artery thrombosis revealed a significant increase in ligand uptake after injection of ^111^In-LIBS in the presence of small thrombi compared to the non-injured side, as confirmed by histology (49630±10650 DLU/mm^2^ vs. 17390±7470 DLU/mm^2^; *P*<0.05). These findings could also be reproduced *in vivo*. SPECT-CT analysis of the injured carotid artery with ^111^In-LIBS resulted in a significant increase of the target-to-background ratio compared to ^111^In-control (1.99±0.36 vs. 1.1±0.24; *P*<0.01).

**Conclusions/Significance:**

Nuclear imaging with ^111^In-LIBS allows the detection of platelet activation *in vitro* and *ex vivo* with high sensitivity. Using SPECT-CT, wall-adherent activated platelets in carotid arteries could be depicted *in vivo*. These results encourage further studies elucidating the role of activated platelets in plaque pathology and atherosclerosis and might be of interest for further developments towards clinical application.

## Introduction

Molecular imaging of cells or cellular epitopes is a rapidly evolving field, which allows non-invasive detection of vascular pathologies [1], [2]. Targeting of surface epitopes in atherosclerosis has been described in various animal models, allowing detection of early atherosclerosis or thrombus formation in arterial vessels [3], [4], [5]. The detection of intravascular thrombosis is still clinically challenging and mostly relies on indirect imaging modalities. Computed tomography angiogram only allows detection of rather large thrombus formation by indicating an altered contrast flow surrounding the thrombosis. Small vessel thrombosis in pulmonary embolism can be detected by scintigraphy, showing a discrepancy between ventilation and perfusion in the suspected area. However, direct targeting of the intravascular thrombosis with molecular imaging would strongly enhance the sensitivity, allowing direct depiction of even smallest intravascular aggregates and specific detection of the underlying pathology. An interesting and clinically relevant target are activated platelets, since they play a key role in atherosclerosis and atherothrombosis [6]. Activated platelets can be found on the surface of inflamed non-ruptured plaques [7], [8], and participate in thrombus formation after plaque rupture [6] leading to myocardial infarction or stroke. Therefore, early and non-invasive detection of platelets in this context would be of clinical interest before total thrombotic occlusion of vessels occurs. Imaging of activated platelets on the surface of arterial thrombosis has been previously described by our group using molecular magnetic resonance imaging (MRI) [8], [9], [10]. As target we addressed ligand-induced binding sites (LIBS), an epitope that is exposed by an activation-specific epitope of the platelet glycoprotein IIb/IIIa-receptor at the site of thrombus formation. In contrast, circulating or resting platelets with an inactive glycoprotein IIb/IIIa-receptor, for example in the spleen, do not reveal these binding sites. Targeting these binding sites with a single-chain antibody that was conjugated to microparticles of iron oxide (MPIO), which typically cause a signal void in T2*-weighted MRI, therefore allowed the selective detection of activated platelets in wall-adherent, non-occlusive thrombosis in carotid arteries of mice in vivo [8]. However, since iron oxide-based contrast agents cause a negative contrast, evaluation of the obtained signal is often disturbed by motion artifacts [11] and small platelet aggregates that are of interest in the context of plaque inflammation could be missed due to lack of sensitivity. Therefore, a different imaging strategy providing improved sensitivity will be needed, especially with the interest of imaging coronary plaque inflammation or rupture in the future. Radiotracers provide an excellent sensitivity, allowing detection down to picomolar concentration [12], [13]. Furthermore, in radiotracer imaging motion artifacts such as the beating heart do not impact sensitivity as severely as in MRI.

Here we describe the construction of a radiotracer specifically targeting the LIBS-epitope of activated platelets in a mouse model of carotid artery injury, which imitates the surface of an inflamed or ruptured plaque. The LIBS-single chain antibody was conjugated to ^111^Indium, and binding to activated platelets tested *in vitro* by autoradiography. In further steps, this approach was transferred to a living system, allowing the detection of thrombosis *ex vivo* by autoradiography and *in vivo* by SPECT-CT. The carotid arteries were identified by CT-angiography, and the images were fused with the ^111^In-LIBS SPECT-examination. This approach allowed the accurate and highly sensitive detection of activated platelets, which is not only of interest for further application in smaller vessels such as the coronary arteries, but also for a future transfer into a human approach.

## Methods

### Ethics Statement

Care and use of laboratory animals in this study followed the national guidelines and was approved by the institutional animal care and ethics committees of the University of Freiburg, Germany (permit No. 35/9185.81/G-09/47).

### LIBS antibody

We used a single-chain antibody that selectively binds to Ligand Induced Binding Sites (LIBS) at the active conformation of the glycoprotein IIb/IIIa receptor and induces strong adherence to activated platelets in the presence of fibrinogen. Antibody construction as well as binding characteristics have been described elsewhere [14], [15]. As control served a similar single-chain antibody, however with a scrambled binding domain, that inhibits specific target binding.

### Coupling of DTPA and labeling with ^111^In

All chemicals were purchased from Sigma-Aldrich (Dreieich, Germany) if not otherwise indicated. ^111^InCl_3_ was obtained from Covidien (Neustadt/Donau, Germany).

The coupling and the labeling were performed in a similar way as described by Ehrenreich et al. [16]. Briefly, the LIBS (max. 200 µg/mL; 200 µg) and the control-scFv (7800 µg/mL; 200 µg) were rebuffered from PBS to an alkine 0.1 M NaHCO_3_ solution with a 10 kDa Amicon Ultra 4 cut-off filter (Millipore Corporation, Molsheim, France). Prior to this, the filter was incubated at 4°C with 1 mg/mL bovine serum albumin (BSA) solution overnight to saturate free protein binding sites.

Afterwards 5 mg DTPA (p-SCN-Bn-DTPA, Macrocyclics, Dallas, TX, USA) was dissolved in the NaHCO_3_ buffer and pipetted onto the filter vial. After incubation for one hour at room temperature the filter vial was filled up with 4 mL NaHCO_3_ buffer and centrifuged once. The incubation step was repeated once.

The DTPA conjugates were rebuffered to 0.1 M NH_4_-acetate buffer (pH 5.4) and three times centrifuged with 4 mL to a final volume of 1 mL. Finally the concentration of DTPA-scAb was determined using Bio-Rad Protein Assay (Bio-Rad Laboratories GmbH, München, Germany) and the extinction was measured on a Spectrometer (SpectraMAX plus, Molecular Devices, Sunnyvale, CA, USA) at 595 nm.

20 MBq ^111^InCl_3_ in 30 µL 0.1 M HCl were added to 40 µg of scFvs in a volume of 600–700 µL ammonia acetate buffer (0,1 M; pH 5,4). For 30 min the sample was incubated at room temperature. Free ^111^In was separated by filtrating it on an Amicon cut-off filter by centrifugation with 4 mL NH_4_-acetate buffer. The radiochemical purity of the ^111^In-labeled scAb was evaluated by running an isocratic HPLC (Ramona Star, raytest GmbH, Straubenhardt, Germany) on a SEC 125-5 Bio-Silect column (Bio-Rad) with PBS as eluent. The ^111^In-labeled LIBS (^111^In-LIBS) and control scFv (^111^In-control) were used for the experiments.

### Functional testing of conjugated antibody with flow cytometry

Persistence of LIBS or control single-chain antibody function after conjugation to DTPA was tested using flow cytometric analysis. Platelet rich plasma was prepared from human whole blood as described elsewhere [16]. Non-activated platelets and platelets activated by adenosine diphosphate (ADP, möLaboratory, Langenfeld, Germany) were examined. After incubation with conjugated LIBS or control single-chain antibody, platelets were exposed to a secondary antibody (Penta HIS Alexa Fluor 488, Qiagen, Hilden Germany) which selectively binds the histidine-tag of the single-chain antibody constructs, and flow cytometry was performed gating 10 000 platelets using a FACSCalibur flow cytometer (Becton Dickinson, Franklin Lakes, NJ, USA). For signal evaluation we used the program CellQuest 3.3 (CellQuest Inc.; Tampa, FL, USA).

### Autoradiography

Autoradiography was conducted using a highly sensitive Phosphor Imaging System (Cyclone Plus, PerkinElmer, Waltham, MA, USA). Results were measured in digital light units per mm^2^ (DLU/mm^2^). DLU is an arbitrary linear unit that describes the intensity of photon emissions released during the scan. Exposure of specimens to the film was conducted in a lead-shielded surrounding to exclude scattered radiation at −20°C. For signal evaluation we set a region of interest (ROI) in the middle of each spot and calculated the mean ligand uptake in DLU/mm^2^.

### *In Vitro* analysis of LIBS functional binding to activated platelets

For *in vitro* studies of ^111^In-LIBS function, cell culture wells were coated with activated or non-activated human platelets. Platelet rich plasma was prepared from whole human blood using sepharose CL-2B and eluted in phosphate buffered saline (PBS). Platelet concentration was determined with a Neubaueŕs counting chamber. Wells (1,9 cm^2^) were pretreated with fibrinogen and blocked with 1% BSA. Then wells were loaded with 200 µl of platelet rich solution (80×10^5^ platelets/well). ^111^In-labeled LIBS or labeled control single-chain antibody, respectively, were incubated on wells, washed three times with PBS at 4°C and submitted to autoradiographic phosphor imaging with different antibody doses as measured by radioactivity in kilocounts per minute (kcpm: 160, 320 and 640 kcpm). Results were evaluated by defining a ROI in the center of the ligand uptake and measuring the corresponding DLU/mm^2^.

### Carotid artery thrombosis model: induction of a wall adherent, non-occlusive thrombosis

For *ex vivo* and *in vivo* experiments we used the well-established carotid artery thrombosis model in mice [5], [17], [18]. Sample size was chosen according to our previous experience with the mouse carotid artery thrombosis model [15], [19]. Ten to 11 week old male C57BL/6 mice (Charles River, Germany) weighing 22±2 g were anesthetized by s.c.-injection with ketamine (200 mg/kg body weight) and xylazine (12.5 mg/kg body weight) and were then placed under a dissecting microscope. A segment of the right carotid artery was exposed through a superficial incision of the skin and blunt dissection of the fascia over the vessel. To obtain a semi-occlusive platelet-rich thrombosis a filter paper (3×3 mm, GB003, Schleicher & Schuell) saturated with ferric chloride (6.5% solution, Sigma, Germany) was placed under the vessel for 3 min. The wound was then closed with a suture of the skin. We conducted an incubation of 45 min to allow appositional growth of the intravascular thrombosis, according to previous experience [5] prior to our scans. After image acquisition animals were terminally anesthetized using ketamine and xyalazine, flushed with saline via the left ventricle, and the injured carotid artery as well as the non-injured contralateral carotid artery were removed.

### *Ex Vivo* experimental protocol

Mice were assigned to either the LIBS or the control group. Due to the restricted half-life of radiotracers this was done in blocks of about 5 animals each. Wall-adherent thrombosis was induced applying 6.5% ferric chloride for 3 min as described above and the operation site was closed with a suture. After 45 min, a venous catheter was placed in the tail vein (Portex, Smiths Medical International, USA) and 100 µl of radiotracers (^111^In-LIBS: 0.98±0.36 MBq, about 10 µg LIBS antibody or ^111^In-control: 0.86±1 MBq, about 10 µg control antibody, respectively) were slowly injected intravenously. Mice were sacrificed 30 min after injection and flushed with physiological saline solution via the left ventricle to clear the vessels from blood. The injured as well as the contralateral carotid were then carefully resected to avoid contamination with tracer remnants, washed and wrapped in plastic foil. At −20°C vessels were placed on the phosphor imaging plate for about 24 hours. The contralateral side served as reference for the assessment of the background noise in each vessel.

### *In vivo* experimental and SPECT-CT protocol

For *in vivo* imaging we again used the carotid artery thrombosis model described above. Compared to ex vivo models, in vivo studies require higher doses of antibody (about 10 fold) to ensure sufficient radioactivity for non-invasive detection of the area of interest. This may hinder a direct comparison between studies, but doses within each study were consistent. Radiolabeled ^111^In-LIBS (16.75±6.28 MBq) or ^111^In-control (13.96±6.97 MBq) was injected via the tail vein catheter. Animals were placed in an animal bed and anesthesia was continuously transferred from ketamine to 1% isoflurane in O_2_.

Scans were performed using a dedicated high resolution small animal SPECT-CT imager (NanoSPECT-CT imager, Bioscan). First, a CT-angiogram of the neck region was conducted for detailed anatomical information of the carotid arteries. The field of view covered 20.4 mm. Images were acquired over 90 sec with 180 projections (exposure time per projection: 500 msec; peak tube voltage: 45 kV; tube current: 177 µA) Using a syringe pump (Harvard Apparatus, Holliston, USA) Imeron 350 iodinated CT contrast (Byk Gulden, Konstanz, Germany) was continuously delivered at a rate of 200 µl/min throughout image acquisition as described elsewhere. [20], [21]. CT images were reconstructed with a resolution of 200 µm.

SPECT analysis of the same area was assessed using a four head system with multi-pinhole collimators (9 pinholes per head, 1.4 mm). Each of 24 projections to cover 360 degree was measured for 600 sec with a total scan time of 60 min. Acquisition time in one case was doubled due to 50% reduction of the activity used (1200 sec and 120 min, respectively). Photopeaks for ^111^Indium were set to 171 keV and 245 keV±5%. SPECT images were reconstructed using the software InvivoScope (Bioscan).

As CT and SPECT images are generated sequentially we realigned images using a three dimensional external fiducial containing 1 MBq of 99m-technetium (^99m^Tc) in a small plastic tubing (Portex, Smith Medical, Ashford, Kent, UK) detectable with CT and firmly fixed to the neck region of the mice. After image reconstruction this three dimensional landmark was used for exact realignment of CT and SPECT images. Photopeaks for ^99m^Tc were set to 140 keV±5%. Images were reconstructed to a voxel size of 300 µm. Finally carotids were harvested for histological workup.

### Evaluation of SPECT-CT images and quantification of signals

Image rendering was conducted using the DICOM viewer Osirix 3.7.1 (Pixmeo, Geneve, Switzerland). Evaluation of SPECT-CT images was done with InvivoScope software (Bioscan).

The ligand uptake, displayed in counts per minute (min), was converted to kilobecquerel (KBq) by defining a quantification factor using a fix amount of ^111^Indium in a water phantom as a reference. For each specimen we correlated the images of the injured vessel with histology (see below) to locate the intravascular thrombosis. We then calculated a ratio of the mean ligand uptake per volume at this area divided by a mean ligand uptake per volume of three surrounding VOIs (Volume of interest) to represent the uptake in the surgical bed. Selection of VOIs based on the CT image only to avoid bias. Single VOIs in the area of the vessel injury had a mean size of 0.68±0.08 mm^3^. The VOIs of the surrounding were chosen with a mean size of 3.63±0.3 mm^3^ to represent a large area to reduce the noise.

### Tissue harvesting and histology

As described previously, animals were terminally anesthetized with ketamine and xylazine after the scans. The injured as well as the contralateral carotid arteries were then removed, embedded in OCT TissueTec (Sakura Finetec, Netherlands) and frozen for histology. For our *ex vivo* experiments the vessels were soaked with OCT and wrapped into plastic for autoradiographic evaluation prior to the final embedding. Frozen tissue was cut in transversal sections of 10 µm at intervals of 30 µm. To assess the location of the thrombosis we identified the bifurcation of the carotid artery as a landmark that could be easily detected by histology and on the rendered CT image. Setting the carotid bifurcation as a benchmark we received a measure for the best placement of the VOI. For the detection and quantification of wall-adherent thrombosis, mouse platelets were detected by immunohistological staining using a rat anti-mouse glycoprotein IIb (CD41) polyclonal antibody (Clone MWReg30, GeneTex, USA). The primary antibody was detected using a rabbit anti-rat biotinylated secondary antibody (Vectastain ABC-AP Kit, Vector, Germany) and VectorRed (Vector, Germany). For each animal, representative sections were chosen and the degree of thrombosis was quantified in percent of the total vessel lumen using Axiovision Software (Carl Zeiss, Germany).

### Statistical methods

All data represent the mean value ± SD. The impact of the results as well as the difference in tracer uptake between the arterial thrombosis and the surrounding were tested via an unpaired, two sided t-test. Test results were considered significant when p<0.05.

The authors had full access to and take full responsibility for the integrity of the data. All authors have read and agreed to the manuscript as written.

## Results

### Labeling and *In vitro* binding of ^111^In-labeled LIBS to activated platelets

**Figure 1:**
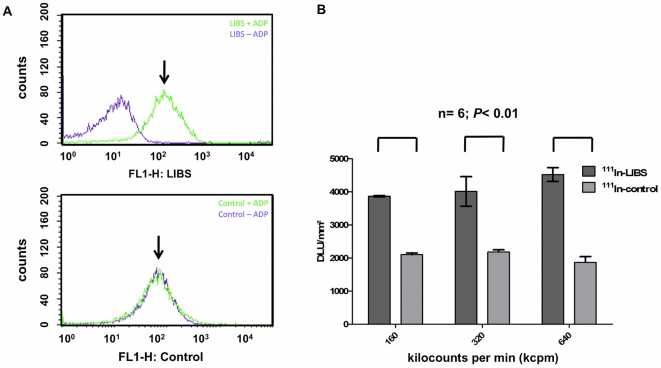
*In vitro* analysis of specific ^111^In-LIBS binding to activated platelets. (A) Flow cytometric analysis of platelets, non-activated (-ADP) and activated (+ADP), after incubation with DTPA-labeled LIBS or control as indicated. After incubation with DTPA-labeled LIBS, activated platelets showed a clear shift indicating specific target binding (above, ↓). This was not seen after incubation on non-activated platelets or application of the control antibody (below, ↓) (B) Autoradiographic imaging of incubated ^111^In-LIBS on activated platelets compared to ^111^In-control. Ligand uptake is depicted in digital light units/mm^2^ (DLU, y-axis) after incubation with increasing antibody doses, as measured by radioactivity (in kilocounts per minute (kcpm); in parenthesis the protein mass used): 160 kcpm (29 ng), 320 kcpm (58 ng) and 640 kcpm (116 ng), respectively (x-axis). A significant increase in ligand uptake is registered after incubation with ^111^In-LIBS (left column) compared to ^111^In-Control (right column) at every activity level (n = 6; *P*<0.05). Given are mean values ± one standard deviation.

The ^111^In-labeled single-chain antibody fragments showed an ^111^In-labeling efficacy of 68.8±8.7%. After purification the radiochemical purity was more than 90%. In vitro experiments with DTPA coupled LIBS antibody using flow cytometry confirmed the persistence of selective binding to activated platelets after conjugation to DTPA (Figure 1A) and isotope labeling (Figure 1B). As shown in Figure 1A, incubation of activated platelets with labeled LIBS results in a relevant shift which is not seen after incubation on rested platelets or incubation with labeled control antibody. Afterwards, three groups with ADP-activated platelets were incubated with either ^111^In-LIBS or ^111^In-control at increasing doses of radiolabeled antibody (160, 320 and 640 kilocounts per minute (kcpm)). By measuring the ligand uptake with autoradiographic imaging, we demonstrated the selective binding of ^111^In-labeled antibodies to activated platelets compared to non-specific binding qualities of ^111^In-control (Figure 1B). At all antibody doses, incubation with ^111^In-LIBS produced a significantly higher ligand uptake (mean 3866±28 DLU/mm^2^, 4010±630 DLU/mm^2^ and 4520±293 DLU/mm^2^) compared to ^111^In-control (2101±76 DLU/mm^2^, 1181±96 DLU/mm^2^ and 1866±246 DLU/mm^2^, *P*<0.05, n = 6).

### *Ex vivo* autoradiographic detection of activated platelets in carotid artery thrombosis

**Figure 2:**
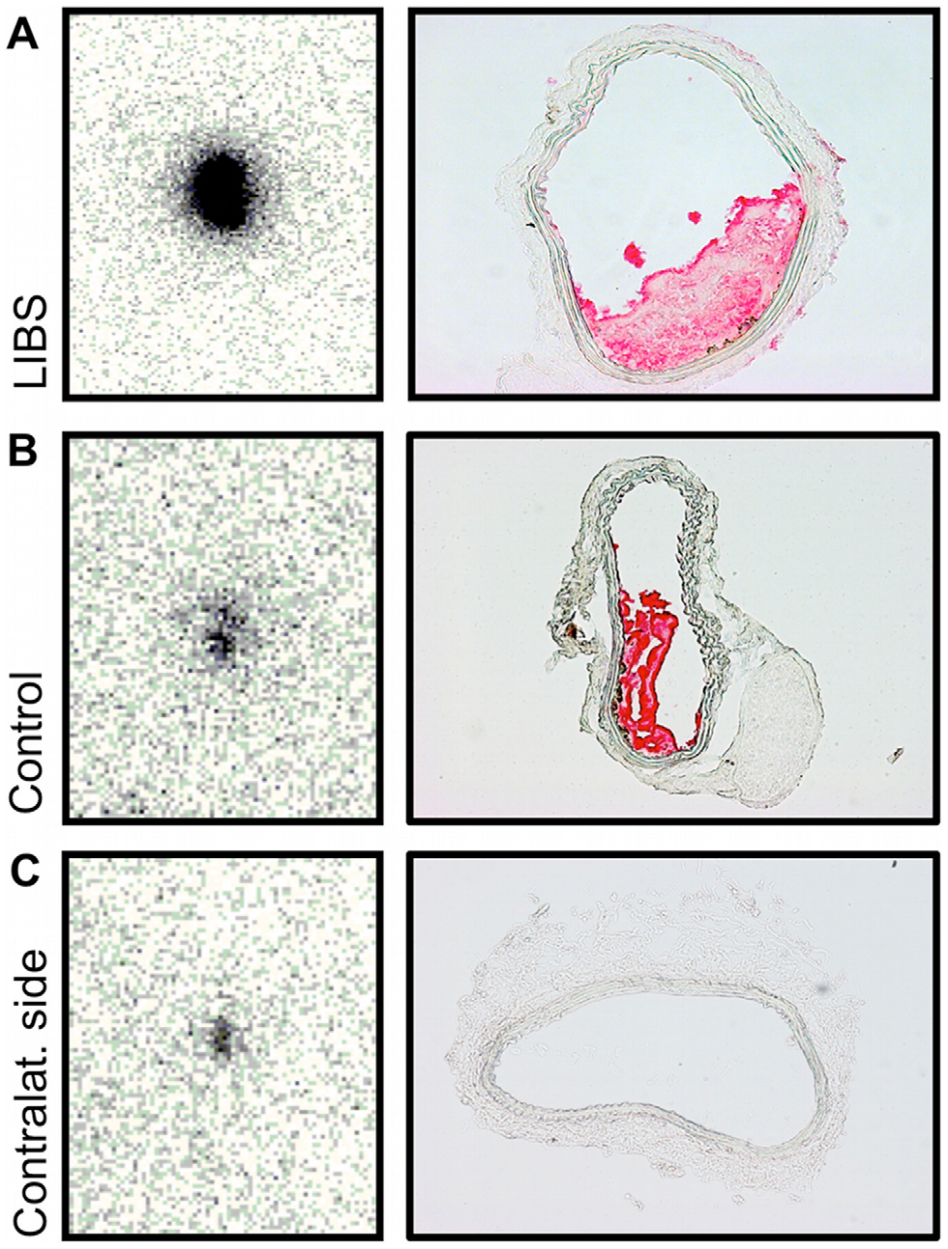
*Ex vivo* autoradiography of carotid artery thrombosis. *Ex vivo* autoradiography of carotid artery specimens (left column) and appendent transversal histology sections of carotid arteries immunohistochemically stained for CD41 (right column). Autoradiography of the injured carotid artery, after treatment with ferric chloride, reveals a strong ligand uptake after incubation with ^111^In-LIBS (A). Corresponding histology proves the presence of a non-occlusive wall-adherent thrombosis. After incubation with ^111^In-control, the radioligand uptake appears visually decreased (B). Autoradiography of the carotid artery on the contralateral non-injured side allowed the assessment of background radiation in the absence of an intravascular thrombosis (C).

A wall-adherent non-occlusive arterial thrombosis was induced in mice and labeled antibodies were injected. This was well tolerated by all animals. After incubation mice were sacrificed, the carotid arteries harvested and analyzed (Figures 2A and 2B). To ensure a relevant but non-occlusive thrombosis, we only selected specimens with a thrombosis >10% and <80% for further data evaluation (mean 40±21%). The degree of thrombosis did not relevantly differ between groups (^111^In-LIBS: n = 5; mean 42±26%, ^111^In-control: n = 5; mean 38±18%).

Next to the evaluation of the injured carotid artery, evaluation of the contralateral non-injured carotid artery served as a negative control and predominantly as reference of remnant radioactivity in the non-injured vessel (Figure 2C).

**Figure 3:**
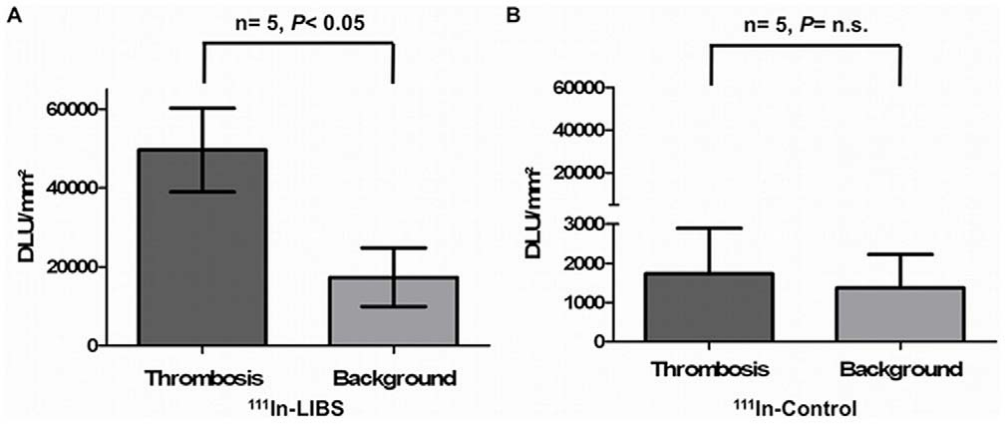
Evaluation of *ex vivo* autoradiographic results. Autoradiographic results after incubation of the injured and non-injured carotid artery with ^111^In-LIBS or ^111^In-Control. Compared to the contralateral non-injured side, indicated as “Background“, incubation with ^111^In-LIBS results in a significant increase in the absolute autoradiographic ligand uptake (DLU/mm^3^) of the injured carotid artery (A; n = 5; *P*<0.05). Changes in uptake were not significant after incubation with ^111^In-control (B; n = 5, p = n.s.). *Ex vivo* results indicate a specificity of ^111^In-LIBS for targeting activated platelets.

Comparing the ligand uptake of the injured with the contralateral reference vessel (n = 5 vs. 5) after incubation with ^111^In-LIBS revealed a significant increase in ligand uptake at the side of the intravascular thrombosis (Figure 3A; 49630±10650 DLU/mm^2^ compared to 17390±7470 DLU/mm^2^, *P*<0.05). In contrast, incubation with ^111^In-control (n = 5) caused no significant elevation of the ligand uptake, but revealed only a subtle signal increase as seen in our *in vitro* study (Figure 3B; 1736±522 DLU/mm^2^ compared to 1373±385 DLU/mm^2^; *P = n.s.*). As the area of interest in the carotid arteries is very small, the initial incubation time on the autoradiographic film was extended to ensure a sufficient signal uptake knowing the risk of image overexposure. This technical issue did however not affect results and was considered not relevant in this ex vivo proof-of-concept. Therefore incubation time was not changed to preserve consistency.

### SPECT-CT imaging of carotid artery thrombosis in mice

**Figure 4:**
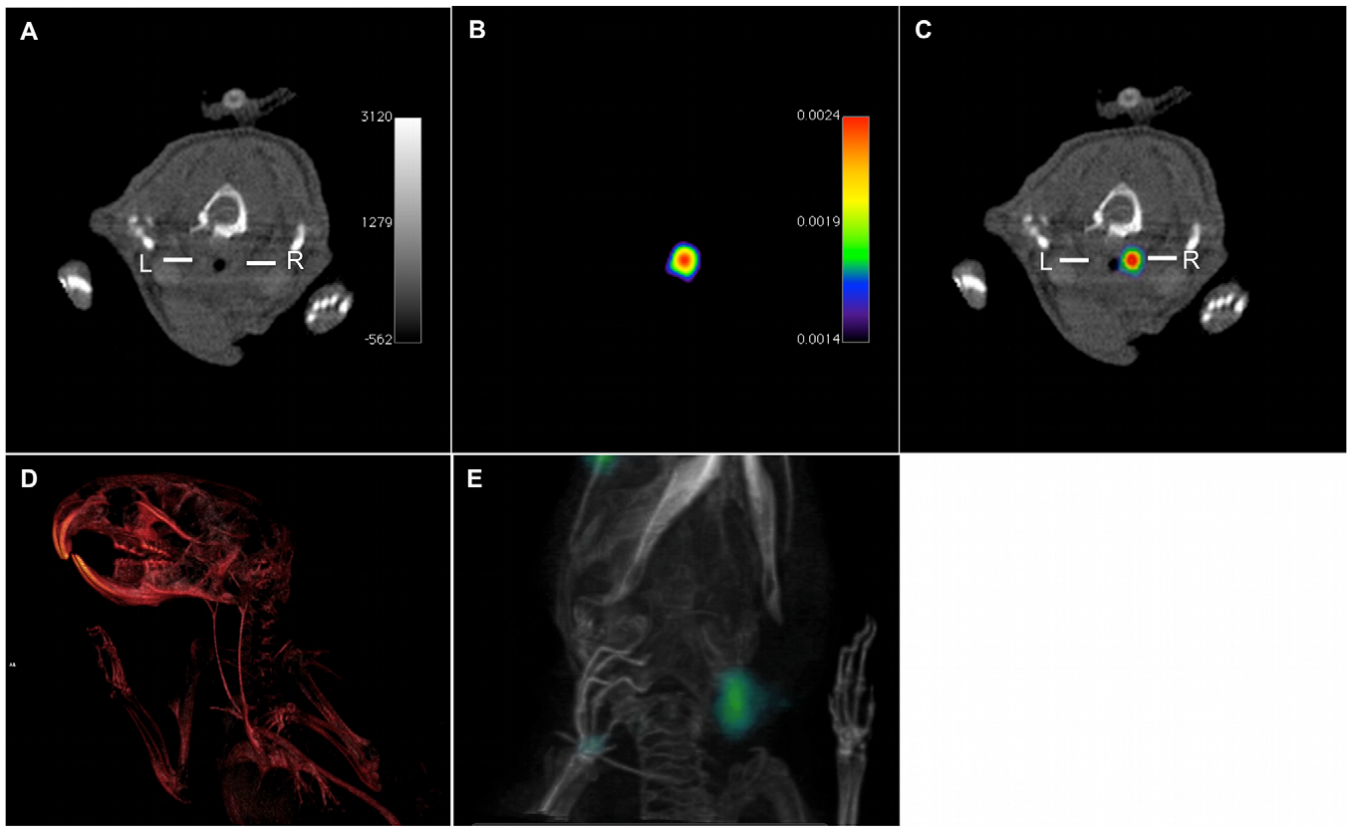
In vivo SPECT-CT imaging of carotid artery thrombosis. *In vivo* SPECT-CT single (A, B) and fused (C) images after injury of the right carotid artery (right side) and incubation with ^111^In-labeled LIBS. CT angiogram of the neck region enhanced with iodinated contrast (Imeron 350) provides vessel contrast for anatomical detail of the carotid arteries (A, R = right carotid vessel, L = left carotid vessel); three-dimensional reconstruction of the CT-angiogram (D). SPECT imaging of the same neck region after incubation with ^111^In-LIBS depicts a co-localized peak of ^111^In-uptake (kBq/voxel). Three-dimensional overlay of CT data (D) with SPECT signal allows the correlation of the peak uptake to the area of the injured carotid artery (E) Movies of the three-dimensionally rendered images are provided as supporting information (Movie S1, Movie S2).

After induction of a thrombus in the right carotid artery, we injected either ^111^In-LIBS or ^111^In-control via a tail vein catheter. Thereafter, mice were placed in the small animal SPECT-CT scanner and carotid arteries were localized using contrast enhanced computed tomography of the neck region. Continuous infusion of Imeron 350 throughout the scan was well tolerated by the animals (Figure 4A). SPECT imaging of the same region was performed without moving the animal (Figure 4B). Prelude whole body biodistribution imaging directly after i.v.-injection showed rapid blood pool clearance of the antibodies with predominantly renal but also low hepatobiliary elimination of tracers (data not shown). Based on this data, SPECT imaging was started 30 min after tracer injection, directly following the CT-angiography.

**Figure 5:**
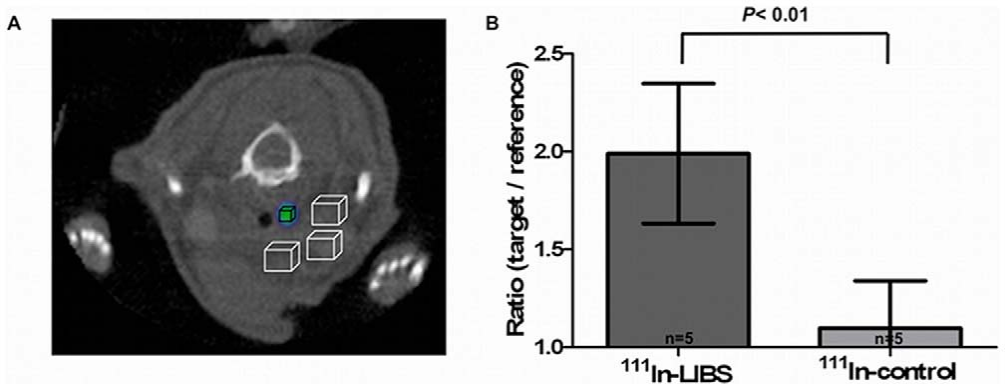
Evaluation of *in vivo* SPECT-CT results. (A) SPECT images were evaluated with reference to the anatomical information provided by CT and based on the information gathered from histological sections about the location of the intravascular thrombosis. To compare the intravascular ligand uptake with the ligand uptake in the surgical bed, we defined 4 VOIs: A small VOI to best represent the target area of the thrombosis (mean 0.68 ±0.08 mm^3^; black box) surrounded by 3 reference VOIs (mean 3.63±0.3 mm^3^) with fixed location to assess ligand uptake in the surgical bed (cubes with an edge length of 1–2 mm; white boxes). From these VOIs we calculated a target to region ratio (i.e., mean uptake per volume in the black box divided by the mean uptake per volume of all white boxes). Comparing the ratio after injection of ^111^In-LIBS and ^111^In-control reveals a significant increase in ligand uptake after incubation with ^111^In-LIBS (B, *P*<0,01).

Exact overlay of images was supported using small external radioactive, CT sensitive three dimensional marker. Figure 4C illustrates the overlay of SPECT and CT data, figures 4D and E represent three dimensionally rendered images of the CT-angiogram and the SPECT-CT. As seen in figure 4C the SPECT ligand uptake visually projects to the site of the vessel injury with a peak uptake on the injured vessel. Uptake was found not only in the injured vessel, but also in the adjacent surrounding (surgical bed) which however was considerably lower. To overcome the interference of ligand uptake at the surgical site with the uptake of the injured vessel, we adopted the surgical bed as background and analyzed a target-to-background ratio to show the increase of ligand uptake after incubation with ^111^In-LIBS compared to ^111^In-control in the area of the vessel injury (as shown in Figure 5A). Incubation with ^111^In-LIBS resulted in a significant increase of this target-to-background ratio (mean ratio 1.99±0.36; n = 4) compared to incubation with ^111^In-control (ratio 1.1±0.24; n = 4; Figure 5B); p<0.01. Ligand uptake of the contralateral non-injured carotid was equivalent to unspecific uptake, for example in the muscle.

## Discussion

Imaging of atherosclerosis and its structural changes in the vessel wall is an emerging field of investigational interest as it provides an approach to early detection of cardiovascular disease. Platelets take part in early as well as in late steps of atherosclerosis and are key players in the development of atherothrombosis [8]. In this study, we describe imaging of activated platelets with a radiopharmaceutical consisting of a single-chain antibody targeting ligand-induced binding sites of the activated fibrinogen receptor glycoprotein IIb/IIIa (α_IIb_β_3_, CD41/CD61) labeled with ^111^Indium.

^111^Indium was chosen for several reasons for the radiolabeling of LIBS. The conjugation with the chelator DTPA is very easy and offers a one pot labeling. The labeling with ^18^F (as a PET nuclide) of proteins is very limited due to a complex chemistry combined with a short half life of the nuclide (110 min). Additionally, ^111^In has good physical properties with a half life of 2.8 days and can be used as a SPECT radionuclide which shows a better resolution in small animals (<1 mm) compared to PET (e.g. ^68^Ga>3 mm). Despite the better sensitivity of PET we chose ^111^In for an initial proof-of-principle due to the higher resolution in small animals and the much easier chemistry combined with a longer half-life.

Previous studies investigating paramagnetic labeled LIBS antibody with magnetic resonance imaging (MRI) have already elucidated the unique binding qualities of this antibody to selectively target activated platelets with high specificity [5], [9], [15]. Using an *in vitro* setup of activated human platelets we confirmed this specific binding to activated platelets with nuclear autoradiographic imaging by showing a significant increase in ligand uptake that occurred exclusively after incubation with ^111^In-LIBS. Therefore, ^111^In-LIBS antibody can be used to selectively depict activated platelets and seems to be suitable for the detection of intravascular thrombosis with nuclear imaging techniques.

To evaluate the potential of ^111^In-LIBS to detect activated platelets on the surface of intravascular arterial thrombosis in an *in vivo* situation, we transferred this contrast agent approach to a mouse model of wall-adherent non-occlusive thrombosis. Others have previously described the imaging of activated platelets with nuclear imaging techniques in the low pressure venous system [22], [23]. However, imaging activated platelets under the high shear stress of arterial flow remains challenging. *Ex vivo* autoradiography allowed the direct assessment of ^111^In-LIBS target binding after exposure to arterial flow conditions and natural elimination from the blood pool with high sensitivity [24]. To guarantee the presence of a relevant thrombosis and to ensure blood flow over its surface for the delivery of sufficient bioavailability of radiotracer, we chose specimens with a relevant but non-occlusive thrombosis as confirmed by histology (10–80% of the total vessel occlusion). After injection of the contrast agent and incubation, both carotid vessels were resected and analyzed with autoradiography. The assessment of the contralateral non-injured side served for the measurement of the remnant background radiation. Non-specific uptake was expected due to the extremely high sensitivity of this autoradiographic approach and was minimized by perfusion of vessels with physiological saline solution. Uptake of the injured carotid artery was, hence, evaluated in the context of the present background radiation. The significant increase in ligand uptake after injection of ^111^In-LIBS compared to ^111^In-control proved a sufficient target binding of ^111^In-LIBS even under arterial flow conditions to allow a highly sensitive detection of activated platelets and intravascular thrombosis in the direct assessment of carotid specimens *ex vivo*.

However, as *in vitro* and *ex vivo* studies are artificial constructs that are not likely to fully cover the complexity of an *in vivo* environment, we used a dedicated small-animal SPECT-CT scanner to evaluate the capability of ^111^In-LIBS to also detect platelet activation and intravascular thrombosis i*n vivo.*

Nuclear imaging techniques such as SPECT provide the possibility of functional analysis of *in vivo* processes, allowing the detection of even small amounts of bound ligands down to picomolar concentration [25]. However the techniques suffer from unclear anatomical localization of the radioactive uptake. This can be overcome by using hybrid imaging, combining SPECT's functional analysis using ^111^In-LIBS for delivery of information about the localization of activated platelets inside the vessel with CT angiography for detailed anatomical information allowing the accurate identification of the carotid arteries.

Since radiotracer-methods provide an excellent sensitivity exceeding the properties of other molecular imaging contrast agents and techniques [12], the combination of these two techniques is a promising approach for characterization of atherosclerosis. Other studies have described successful application of SPECT-CT for the characterization of atherosclerosis. Using a radioligand against the oxidized low-density lipoprotein receptor, molecular imaging of atherosclerotic plaques was possible in a murine model, and imaging signal was associated with markers of rupture-prone atherosclerotic plaques [26]. Also evaluation of atherosclerosis by *in vivo* SPECT-CT has been described, targeting annexin and matrix-metalloproteinase inhibitors [27], [28]. Also the activity of matrix-metalloproteinases in atherosclerotic lesions of New Zealand rabbits was detectable by SPECT and allowed monitoring of dietary modification and statin treatment [29]. However, to our knowledge, imaging of platelets has not yet been described in the context of atherothrombotic diseases. Sensitive and specific imaging would be of interest, since platelets can be found on the surface of inflamed, rupture-prone plaques and are early indicators of plaque rupture [6], [7], [8]. These properties exceed the advantage obtained by imaging of other thrombus components, such as described with fibrin or D-dimer antibodies, which have been recently used to image pulmonary emboli by SPECT [30].

By showing a significant increase in the target to background ratio after injection of ^111^In-LIBS by SPECT that could be well correlated with the presence of a relevant arterial thrombosis, we were able to detect platelet activation in advanced atherothrombotic disease. Having proven the concept of this approach, next steps will now use the high sensitivity of nuclear imaging techniques to further investigate the surface of inflamed endothelium and rupture prone plaques to identify and elucidate the role of platelets in inflammatory processes and the development of atherosclerosis.

Furthermore, direct non-invasive targeting of intravascular thrombosis would be a unique and novel approach to detect arterial, but also venous thrombosis at much higher sensitivity compared to the known imaging techniques such as CT angiography or ventilation-perfusion scintigraphy. The prerequisites for a bench to bedside transfer of LIBS-scFv are excellent. LIBS-scFv was initially designed for application on human platelets acting as activation-specific antagonist of their gpIIb/IIIa-receptor. ScFvs are a small functional form of an antibody, only consisting of the variable regions of the antibody's heavy and light chain fused together via a short linker molecule, and can be produced in bacteria at low costs [31], [32]. The specificity of its target binding is unique, and target affinity is comparable to clinically used gpIIb/IIIa antagonists. The small size of the LIBS-scFv (about 32 kDa) allows for good accessibility and penetration of the thrombus [32] as well as rapid blood pool clearance via the kidney of non-bound labeled protein. Fortunately, cross-reactivity with mouse platelets allows the assessment of the LIBS-antibody as a probe for molecular imaging in mouse models. Nuclear imaging techniques have been used for years to detect molecular receptors in oncology [33] and encounter broad clinical acceptance. Therefore, nuclear imaging of activated platelets would be a first step towards clinical application. A transfer from bench to bedside would be certainly challenging but worthwhile with regards to the therapeutic benefits. While the risk of immunogenicity is extremely low [31], selective targeting of activated platelets provides pathophysiologic information which allows for individual risk stratification and will help to guide therapeutic strategies. Favorable non-radioactive molecular imaging techniques such as MRI with MPIOs for the detection of activated platelets have also been evaluated by our group [5]. These techniques are certainly promising, however have not yet reached the level of clinical applicability due to potential toxicity of the contrast-giving molecules.

A possible limitation of the animal model applied in our study for further functional evaluation of ^111^Indium-LIBS is the need of the carotid artery to be directly exposed for the reliable and reproducible induction of wall-adherent thrombosis. Thereby, a wound area reaching from the skin surface towards the artery is created, allowing non-specific radiotracer deposition in edematous tissue but also specific binding to activated platelets involved into hemostasis. This is the reason for the high signal background in the wound area seen in both animal groups, after injection of ^111^In-control but also with ^111^In-LIBS. However, the uptake signal caused by specific binding of ^111^In-LIBS at the carotid artery thrombosis is still sufficient to obtain a highly significant signal. The reason for applying this model in our study in spite of these disadvantages is its reproducibility [5], which is an important prerequisite in a proof-of-concept-study. We are currently evaluating alternative animal models to overcome this limitation.

### Conclusions

We describe the construction of a radioligand based on a single-chain antibody specifically targeting activated platelets in an *in vivo* mouse model of wall-adherent non-occlusive thrombosis in the carotid artery, which imitates the surface of an inflamed or ruptured plaque. In all approaches applying *in vitro*, *ex vivo* and *in vivo* techniques, the ^111^In-LIBS radiotracer enabled the sensitive detection of wall-adherent activated platelets, such as found in atherothrombosis or plaque inflammation. SPECT-CT allowed the identification of the carotid artery as well as the accurate and highly sensitive detection of wall-adherent activated platelets. These results encourage further studies elucidating the role of activated platelets in plaque pathology and atherosclerosis and are of interest for future developments towards clinical application since the timely detection of platelet activation on the vessel wall could allow for the individual risk assessment in cardiovascular disease.

## Supporting Information

Movie S1 **Three-dimensional rendering of the CT angiogram.** *Ex vivo* three-dimensional rendering of the CT angiogram provides detailed anatomical information of the carotid arteries' location.
(MOV)

Movie S2 **Three-dimensional rendering of the SPECT-CT.** *In vivo* tree-dimensional rendering of the SPECT-CT after carotid injury and incubation with ^111^In-LIBS. Ligand uptake projects to the area of the carotid injury. Additionally, some ligand uptake can also be found in the orbita of the contralateral side, which is due to contrast agent deposition in the Harder's gland.
(MOV)
